# Effects of Loquat Juice Addition on Sensory Characteristics and Volatile Organic Compounds of Loquat Beer

**DOI:** 10.3390/molecules29163737

**Published:** 2024-08-07

**Authors:** Junjie Li, Lang Li, Pinglian Yu, Banglei Zhang, Lina Zhao, Zhongxia Zhao, Kunyi Liu, Kaijie Kang

**Affiliations:** 1 School of Chemistry and Chemical Engineering, Zhaotong University, Zhaotong 657000, China; lijunjie900501@163.com (J.L.); zhaozx621@163.com (Z.Z.); 2 Key Laboratory for Plateau Characteristic Functional Food Research of Universities in Yunnan Province, Zhaotong, 657000, China; 3 School of Wuliangye Technology and Food Engineering, Yibin Vocational and Technical College, Yibin 644100, China

**Keywords:** loquat juice, beer, sensory characterization, volatile components, physicochemical properties

## Abstract

Beer, as an ancient and widely consumed alcoholic beverage, holds a rich cultural heritage and history. In recent years, fruit beer has gained significant attention as a distinct beer type produced by incorporating fruit juice into traditional beer ingredients. This study employed headspace solid-phase microextraction–gas chromatography–mass spectrometry techniques, redundancy analysis, and orthogonal projections to latent structures discriminant analysis to analyze the sensory evaluation, physicochemical properties, organic acids, and volatile organic compounds (VOCs) of loquat beer with different proportions of loquat juice. The results shown that the addition of an appropriate amount of loquat juice (40%) enhanced the overall sensory quality of the beer; as the proportion of loquat juice increased, the contents of malic acid and tartaric acid significantly increased (*p* < 0.05). A total of 100 VOCs were identified, among which 23 key VOCs (VIP > 1, *p* < 0.05) represented the most important characteristic flavor components in loquat beer based on their odor activity value (OAV). This study holds significant importance for the value-added processing and economic development of loquat.

## 1. Introduction

Beer, as an ancient and widely consumed alcoholic beverage, is produced through the fermentation of ingredients such as water, malt, hops, and yeast, and holds a long history and rich cultural background [1]. Traditional beers typically exhibit a certain level of bitterness and malty aromas, with variations in taste and flavor attributed to different malt and yeast varieties [2]. Beer enjoys global popularity, with a wide range of styles and varieties available. However, in recent years, there has been an increasing demand for new flavors and diversified beverages, leading to growing interest in fruit beers as a unique beer category. Fruit beers are a special type of beer produced by incorporating fruits or fruit juices into traditional beer ingredients [3,4]. By combining fruits with malt, hops, and yeast, fruit beers showcase rich fruit aromas, taste, and flavors [5]. Some reported fruit beers include loquat beer [6], strawberry beer [7], citrus beer [8], blueberry beer [9], grape beer [10], persimmon beer [11], among others, each offering unique flavor characteristics to the beer. The brewing of fruit beers has been an enduring tradition. The use of various fruits to flavor and ferment beer has remained a popular brewing practice across different cultures and regions. By adjusting parameters such as fruit addition, fermentation time, and temperature, they have sought to find the optimal balance between different fruits and beer. These studies not only expand the flavor range of fruit beers but also provide theoretical and practical guidance for brewing fruit beers with stable texture and distinctive flavors.

Among the various fruit beer varieties, loquat beer has emerged as a distinctive product. Loquat fruits are characterized by their orange-yellow color, soft and juicy texture, pleasant sweet and sour taste, and unique flavor [12]. Bang and Mujinda have demonstrated that loquat fruits are rich in nutrients, including amino acids, proteins, carbohydrates, minerals such as iron, calcium, phosphorus, and various vitamins such as vitamin A, B, and C [13,14]. Moreover, loquat has medicinal value, including cough suppression and gastric protection effects, and is mainly used to treat coughs, asthma, and pediatric fever, earning it the reputation of a health fruit [15,16]. However, due to the short harvesting season and challenges in preserving, transporting, and storing loquats, farmers face significant difficulties and experience substantial losses each year, making value-added processing of loquats necessary [17]. According to reports, fresh loquat contains abundant characteristic volatile organic compounds such as butyraldehyde, 3-methylbutanal, (E) -2-hexenal, 2-butanone, valeric acid, and 4-ethylphenol, and the tartaric acid, malic acid, lactic acid, and acetic acid in loquat play an important role in the taste of loquat [18]. Loquat beer is a low-alcohol beer beverage produced by adding different proportions of loquat juice to malt juice, followed by fermentation. It not only possesses unique flavor characteristics but also retains the nutritional components present in the fruit. These characteristic flavor compounds in fresh loquat combine with the bitter compounds in hops to produce a unique flavor [19,20].

The study of loquat beer holds significant economic value and promising prospects for the in-depth exploration of loquats. In this study, headspace solid-phase microextraction (HS-SPME) coupled with gas chromatography–mass spectrometry (GC-MS) was employed to analyze the volatile organic compounds (VOCs) in loquat beers with different proportions of loquat juice. The types of VOCs were determined, and through the calculation and analysis of odor activity values (OAV), the primary characteristic VOCs in loquat beer were identified. These studies, through sensory evaluation and flavor component analysis, reveal the contribution of loquats to the aroma, balanced taste, and overall flavor of the beer.

## 2. Results

### 2.1. Sensory Analysis Results

**Figure 1:**
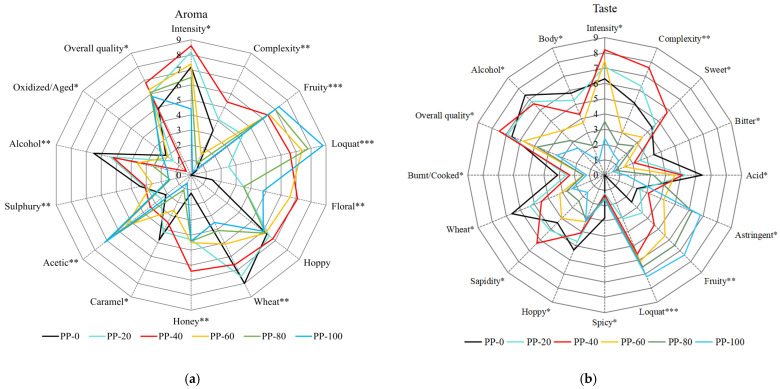
Sensory analysis of loquat beer ((**a**): aroma, (**b**): taste); symbols: ***, *p* < 0.001; **, *p* < 0.01; *, *p* < 0.05.

According to the sensory evaluation from the aroma perspective of the loquat beer, as shown in Figure 1a, the ratings of intensity, complexity, floral, hoppy, honey, and overall quality increase with the addition of loquat juice. The highest rating is achieved when the loquat juice is added at 40% (PP-40). However, as the loquat juice content exceeds 40%, the ratings gradually decrease. The ratings of fruity, loquat, and acetic also increase with the increase in loquat juice content. On the other hand, the ratings of wheat, caramel, sulphury, and alcohol decrease as more loquat juice is added. From the taste perspective, as depicted in Figure 1b, the ratings of intensity, complexity, sweet, sapidity, and overall quality increase with the addition of loquat juice. The highest rating is observed at 40% loquat juice content (PP-40). However, when the loquat juice content exceeds 40%, the ratings gradually decline. The addition of loquat juice leads to an increase in the ratings of astringent, fruity, and loquat, but it also decreases the ratings of bitter, acid, hoppy, wheat, burnt/cooked, body, and alcohol.

### 2.2. Physicochemical Analysis

Table 1 shows the physicochemical parameters of different loquat beers. The addition of loquat juice decreases the pH, alcohol content, density, and specific gravity of the beer. The beer without the addition of loquat juice has the highest values for pH (4.14), alcohol content (4.85% vol), density (1024 kg/m^3^), and specific gravity (1030 °P). Loquat juice is a fruit juice that typically contains high levels of moisture and natural sugars while also having some acidity. In comparison, malt extract is a raw material used in brewing beer and contains significant amounts of maltose and starch, resulting in higher total sugar content that is favorable for fermentation. The starch in malt extract is converted into alcohol and carbon dioxide by yeast during the brewing process. However, when loquat juice is added, although its natural sugars can be fermented by yeast and increase the total solute content in the solution, the high moisture content in loquat juice dilutes the alcohol concentration in the malt extract, thereby reducing the beer’s specific gravity and density. Additionally, the acidic components in loquat juice, which cannot be utilized by yeast, remain in the beer, resulting in a decrease in pH.

### 2.3. VOCs Content Analysis

As shown in Table 2, a total of one hundred VOCs were detected in all loquat beer samples, including thirty-five esters, thirty-one alcohols, ten aldehydes, nine alkenes, seven ketones, four phenols, and four organic acids. The loquat beer with the highest total volatile compound content is PP-40, with 1045.44 mg/L, primarily composed of esters (20.91%), alcohols (54.48%), and alkenes (16.76%). The loquat beer with the lowest total volatile compound content is PP-100, with 507.16 mg/L, even lower than the beer without loquat juice (PP-0). When loquat juice is added, the total volatile compounds in the beer increase. However, after exceeding a 40% addition rate, the total volatile compounds start to decrease. Among all the samples, alcohols account for 31.15% to 63.25% of the total volatile compounds and are the most abundant compounds in loquat beer. The main alcohols detected include phenylethyl alcohol, 3-methyl-1-butanol, 2-methyl-1-butanol, 2-methyl-1-propanol, 1-nonanol, and geraniol. Phenylethyl alcohol and 3-methyl-1-butanol have the highest concentrations, ranging from 7.35% to 46.87% and 638% to 12.71% of the total volatile compounds, respectively. Phenylethyl alcohol reaches its highest concentration in the PP-60 sample, with 621.97 mg/L, while 3-methyl-1-butanol reaches its highest concentration in the PP-40 sample, with 220.21 mg/L.

### 2.4. Analysis of OAV for Loquat Beer Aroma

The contribution of each VOCs in loquat beer to the main aroma components was analyzed by OAV, and the OAV of each VOCs was calculated by compound concentration and threshold. When the OAV ≥ 1, it can be considered that the volatile matter has a contribution to the overall flavor of the loquat beer sample. It is generally believed that VOCs with high concentration and low olfactory threshold in food are the characteristic aroma components in food [21]. It can be seen from Table 3 that fifty-five VOCs with OAV ≥ 1 in loquat beer were identified, including esters (twenty-two VOCs), fifteen kinds of alcohols, eight kinds of aldehydes, five kinds of alkanes, three kinds of ketones, and two kinds of phenols. The OAVs of compounds in different loquat beer were significantly different, and the OAVs of various VOCs were positively correlated with their concentrations. The higher the OAV, the greater the contribution rate of the volatile compound to the aroma components of loquat beer. However, according to research reports, the greatest contribution to flavor in food does not depend on the concentration [22]. The concentrations of methyl 2-methylbutyrate, ethyl isobutyrate, ethyl butyrate, ethyl 2-methylbutyrate, ethyl heptanoate, γ-decalactone, geraniol, 1-hexanol, linalool, 1-dodecanol, 1-hetanol, 1-octen-3-ol, naphthalene, myrcene, benzeneacetaldehyde, β-cyclocitral, 1-octen-3-one, β-lonone, ethyl benzoate, and eugenol were very low. However, it showed a large OAV, indicating that these substances are important aroma substances that constitute loquat beer.

### 2.5. Correlation Analysis of Key Flavor Compounds, Sensory Evaluation, and Physicochemical Indicators in Loquat Beer

**Figure 2:**
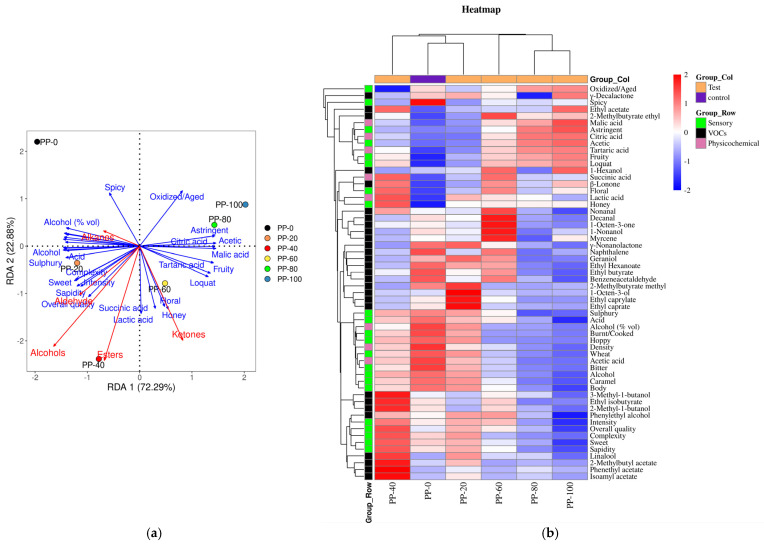
Correlation analysis of VOCs in loquat beer (**a**) RDA plot; (**b**) correlation clustering heatmap.

The key VOCs with OAVs ≥ 1 from Table 3 were selected, and redundancy analysis (RDA) and correlation clustering analysis were performed. As shown in Figure 2a, the red arrows represent the types of VOCs, and the length of the arrows connecting them to the origin represents the degree of correlation between the compound type and sample distribution. Longer arrows indicate stronger correlations, while shorter arrows indicate weaker correlations [23]. Alcohols, esters, ketones, and aldehydes, the four categories of volatile compounds, showed significant correlations in loquat beer, and they exhibited positive correlations with each other, while they showed negative correlations with alkanes. The oxidized/aged and spicy attributes are negatively correlated with esters, alcohols, aldehyde, and ketones. By projecting the sample points vertically onto the red arrows, it can be observed that alcohols, esters, ketones, and aldehydes have the strongest correlations with the PP-40 beer. In terms of sensory evaluation scores, complexity, sweet, sapidity, intensity, overall quality, floral, honey, succinic acid, and lactic acid showed high positive correlations with these four categories of volatile compounds, while they exhibited negative correlations with alkanes. Alkanes showed high positive correlations with spicy, alcohol (%vol), body, acid, sulphury, and complexity.

From Figure 2b, it can be seen that different types of VOCs have significant correlations in different food processing processes. Each row in the heatmap represents a category of VOCs, each column represents a different sample, and the color of each cell indicates the correlation of VOCs in different samples. Deeper red indicates higher correlation, while deeper blue indicates lower correlation. Clustering of rows and columns reveals the similarities and correlations between samples or features [24]. The sensory evaluation scores of sulphury, acid, alcohol (%vol), burnt/cooked, hoppy, density, wheat, acetic acid, bitter, caramel, body, and spicy showed positive correlations in beers without added loquat, but the correlations weakened with the addition of loquat juice. However, tartaric acid, 1-hexanol, β-lonone, lactic acid, succinic acid, fruity, loquat, floral, and honey showed negative correlations in beers without added loquat, but the correlations became stronger with the addition of loquat juice. Among the volatile compounds, 3-methyl-1-butanol, ethyl isobutyrate, 2-methyl-1-butanol, phenylethyl alcohol, linalool, 2-methylbutyl acetate, phenethyl acetate, isoamyl acetate, as well as intensity, overall quality, complexity, sweet, and sapidity in sensory evaluation scores showed high positive correlations in the PP-40 beer sample.

### 2.6. Analysis of Key VOCs in Loquat Beer Based on OPLS-DA

**Figure 3:**
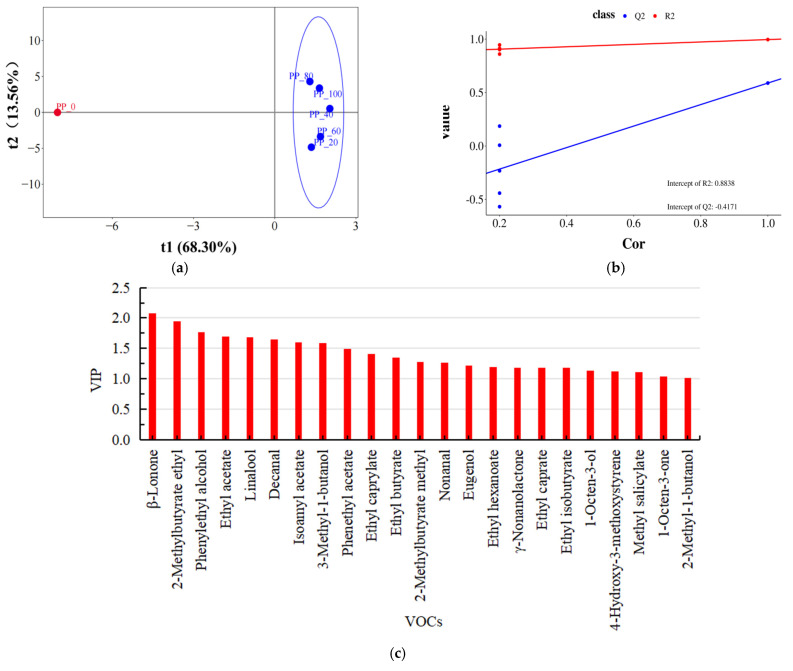
OPLS-DA analysis of key flavor substances in different loquat beer. (**a**): OPLS-DA score plot, (**b**): OPLS-DA permutation test model (R^2^: model explained variance, Q^2^: model predictive ability), (**c**): VIP plot.

Using the orthogonal partial least squares discriminant analysis (OPLS-DA) method, the distribution characteristics of loquat beer were analyzed [25]. As shown in Figure 3a,b, after 200 permutation tests and 7-fold cross-validation, the model obtained an R^2^ value of 0.8838. The intercept of the Q^2^ regression line with the y-axis is less than 0, indicating that the model is not overfitting and the validation of the model is effective. Therefore, the results of this model can be applied to the analysis of key aroma components in loquat beer. The VIP value (Variable Importance in Projection) indicates the importance of a compound in the aroma substances of loquat beer. Generally, a VIP value greater than 1 and a *p*-value less than 0.05 indicate a high contribution of the variable to sample differentiation [26]. From Figure 3c, it can be observed that 23 key VOCs were selected, including β-lonone, 2-methylbutyrate ethyl, phenylethyl alcohol, ethyl acetate, linalool, decanal, isoamyl acetate, 3-methyl-1-butanol, phenethyl acetate, ethyl caprylate, and others. These compounds are also present in Figure 2b, indicating their strong correlation with organic acids. These 23 key VOCs are the most important characteristic flavor components in loquat beer.

## 3. Discussion

Overall, the research findings indicate that, among different sensory indicators, the beer with the addition of 40% loquat juice (PP-40) obtained the highest overall score, exhibiting a rich malt aroma and distinct loquat flavor, with a smoother taste. Conversely, the beer with 100% loquat juice (PP-100) received the lowest evaluation, indicating that adding more loquat juice does not necessarily result in better outcomes. The addition of loquat juice enriches the complexity of the beer’s flavor, but the combination of different flavors can also generate interactions [27]. The studies have shown that the perception and judgment of flavor characteristics in fermented beverages are influenced by multiple factors, which can have antagonistic or synergistic effects, leading to what is known as the “matrix effect” [28,29].

Comparing the samples with the addition of loquat juice (PP-0), the content of succinic acid, malic acid, lactic acid, tartaric acid, and citric acid in the beer increased, while the content of acetic acid gradually decreased. Organic acids are important flavor components in alcoholic beverages and serve as significant indicators for evaluating beer quality [30]. According to the research reports, in fruit fermented beverages, apple acid, tartaric acid, and citric acid mainly come from the raw materials, while lactic acid, succinic acid, and acetic acid are produced as metabolic byproducts of microbial fermentation [31]. Therefore, it can be inferred that the main organic acids in loquat beer are lactic acid, malic acid, and citric acid. The addition of loquat juice not only enriches the content of malic acid, tartaric acid, and citric acid in the beer from the raw materials but also influences the fermentation by micro-organisms, resulting in increased production of organic acids such as lactic acid, succinic acid, and acetic acid.

In addition, regarding the analysis of volatile compounds, previous studies have reported that Pirrone [6] used yeast fermented loquat beer obtained from a sugar-rich source and found that 3-methyl-1-butanol and phenylethyl alcohol were the most abundant volatile organic compounds (VOCs), which is consistent with the results of this study. However, Francesca [32] found that phenylethyl alcohol and 1-pentanol were the most abundant alcohol compounds in loquat beer, which may be related to the use of different yeast strains for fermentation. Esters accounted for 10.33% to 20.91% of the total volatile compounds in all samples, mainly composed of ethyl acetate, ethyl caprylate, phenethyl acetate, ethyl caprate, isoamyl acetate, γ-nonanolactone, ethyl hexanoate, diisobutyl phthalate, 2-methylbutyl acetate, and ethyl laurate. With the addition of loquat juice, the content of ethyl acetate, ethyl caprylate, phenethyl acetate, ethyl caprate, isoamyl acetate, and 2-methylbutyl acetate increases, reaching the highest level at 40% addition (PP-40). However, after exceeding 40% addition, the content of these compounds decreases. The addition of loquat juice significantly reduces the content of γ-nonanolactone, ethyl hexanoate, and ethyl laurate in the beer. Ethyl acetate, ethyl caprylate, and phenethyl acetate are the main aroma substances in loquat beer, which is consistent with the study by Francesca [32]. Esters not only directly affect the aroma of beer but also interact with other compounds in complex ways. Additionally, the fermentation process greatly affects the total ester content [33]. Alkanes accounted for 9.30% to 17.23% of the total volatile compounds in all samples, mainly composed of 1-chloro-pentane and 2,4-dimethyl-1-hexene. The content of these two compounds increases with the addition of loquat juice and reaches the highest level at 40% addition, but it decreases when the addition exceeds 40%. Aldehydes accounted for 0.66% to 1.46% of the total volatile compounds in all samples, mainly composed of decanal and nonanal. Ketones accounted for 0.28% to 1.31% of the total volatile compounds in all samples, mainly composed of 6,10-dimethyl-5,9-undecadien-2-one and 2-hydroxy-3-pentanone. Organic acids accounted for 0.88% to 2.01% of the total volatile compounds in all samples, mainly composed of decanoic acid, isovaleric acid, heptanoic acid, and nonanoic acid. The addition of loquat juice does not have a consistent impact on these compounds. Some aldehydes in addition to being metabolic byproducts of micro-organisms also come from fresh loquats [34]. According to reports, in the later stages of loquat beer fermentation, acids and alcohols interact, resulting in the formation of ester compounds with distinctive aromas. Therefore, the ester content increases in the later stages of loquat beer fermentation [35]. Phenols accounted for 5.70% to 26.45% of the total volatile compounds in all samples. The addition of loquat juice also had a significant effect on the content of 2,5-di-tert-butylphenol, which ranged from 5.62% to 26.21% in all beer samples, with the highest concentration found in the PP-60 sample, reaching 171.57 μg/mL. Phenolic compounds are secondary metabolites of yeast [36], and Francesca’s loquat beer study also detected 2,4-di-tert-butylphenol, which is an isomer of 2,5-di-tert-butylphenol and 2,6-di-tert-butylphenol found in this study. The analysis of OAV for volatile compounds demonstrates that most esters generally contribute to floral and fruity aromas. Alcohols are associated with the aromas of wine, fruits, grass, and fatty substances. Alkanes tend to exhibit notes of charred flavor, herbal scents, and aromatics. Aldehydes are typically linked to fruity, floral, and honey-like aromas. Ketones are often characterized by creamy, fatty, and floral fragrances. Phenols commonly display aromas of spices, flowers, and fragrant herbs. These compounds contribute significantly to the overall aroma of loquat beer and contribute to its unique flavor. Numerical analysis reveals that the OAVs of most VOCs in the PP-40 sample are significantly higher than in other samples, indicating that loquat beer brewed with 40% loquat juice has a more pronounced aroma. Finally, the RDA, correlation clustering heatmap analysis, and OPLS-DA analysis of the 55 volatile compounds with higher OAVs show that there are certain positive or negative correlations among different VOCs. Through VIP analysis, the most important 23 characteristic flavor components in loquat beer are identified.

## 4. Materials and Methods

### 4.1. Strains and Culture Methods

The yeast strain used in this study was Saccharomyces cerevisiae CN36, obtained from Fermentis in France. The yeast was reactivated following the method described by Pirrone (2022) [6]. It was initially cultured in YPD broth at 28 °C for 24 h to increase the cell population. The yeast cells were then collected by centrifugation and washed with a phosphate-buffered saline (PBS) solution (pH 7.0) to remove the residual YPD medium. The yeast cells were resuspended in PBS buffer to obtain a cell suspension.

### 4.2. Experimental Design

Barley malt was crushed and screened using Tyler sieves of 40 mesh and 80 mesh. The oversize particles from the sieving process were collected as crushed barley malt, with 70% by weight from the 40-mesh fraction and 30% from the 80-mesh fraction. The differently crushed barley malt was mixed and added to 3.5 times the volume of drinking water. The mixture was heated to 50 °C and held for 60 min, then raised to 65 °C and held for 30 min. Subsequently, the temperature was further raised to 72 °C and held for 20 min, followed by an increase to 78 °C and a 10 min hold, until complete sugar conversion occurred [37]. The resulting malt extract, with a pH of 5.00 and a sugar content of 11 °Bx (Brix degree) was collected.

The fruits of “Chinese Golden Loquat” harvested from a local orchard (103°51′47″ E to 104°45′4″ E, 27°47′35″ N to 28°17′42″ N) were used to prepare loquat juice according to the method described by Pirrone [6]. The fruits were extracted with sterile water in a ratio of 3:1 (water/fruit) to obtain loquat juice. To prevent discoloration, the juice was mixed with a solution containing 0.5% ascorbic acid and 0.5% citric acid. Pectinase (120 mg/kg) was added to the solution, followed by enzymatic hydrolysis at 38 °C for 3 h. The enzyme was then inactivated by heating at 80 °C for 5 min to obtain the prefermentation loquat juice.

Different proportions of the loquat juice were added to the malt extract to prepare the fermentation substrates for loquat beer, designated as PP-0 (no loquat juice), PP-20 (20% loquat juice), PP-40 (40% loquat juice), PP-60 (60% loquat juice), PP-80 (80% loquat juice), and PP-100 (100% loquat juice). The yeast cells were inoculated at approximately 1.0 × 10^7^ cells/mL, based on the total volume of loquat juice and malt extract. The fermentation was carried out at 10 °C for 15 days [28]. After this period, sensory analysis was performed on the beer, and samples were collected for further analysis. All fermentation experiments were conducted in triplicate.

### 4.3. Sensory Evaluation

A total of 20 judges (10 males and 10 females, students and staff each account for half) aged between 24 and 40 years were invited from Zhaotong University to evaluate the loquat beer. All judges had previous experience in tasting and evaluating beers. Prior to evaluating the loquat beer, the judges underwent preliminary training, and the sample numbers were kept confidential [32]. The sensory evaluation of the loquat beer was conducted following the methods described by Marconi [38] and ISO standards. A total of 22 sensory attributes were determined for evaluating the loquat beer in terms of appearance, aroma, taste, and overall quality. These attributes included intensity, complexity, fruity, loquat, floral, hoppy, wheat, honey, caramel, acetic, sulphury, alcohol, oxidized/aged, overall quality, sweet, bitter, acid, astringent, spicy, sapidity, burnt/cooked, and body. The judges rated each attribute on a scale from 0 to 9, with higher scores indicating a stronger correlation with the specific attribute. The average of the three ratings was considered the final score for each attribute and used for further analysis.

### 4.4. Physicochemical Properties

The pH of the loquat beer was measured using a pH meter (model ST2100 F, AOHUA Instrument (Shanghai) Co., Ltd., Shanghai, China). The determination of butyric acid, malic acid, lactic acid, acetic acid, tartaric acid, and citric acid followed the method described by Matraxia [39]. The alcohol content, density, and specific gravity were measured following the method described by Francesca [32].

### 4.5. Determination of VOCs

The VOCs in the beer samples were determined following the method reported by Zhang [40], but the pre-treatment method of the sample was based on the methods of Piergiovanni [41,42]. A gas chromatography–mass spectrometry (GC-MS) system (model 7890 B-5977 A, Agilent Technologies, Santa Clara, CA, USA) equipped with a DB-5 capillary column (60 m × 0.25 mm, 0.25 μm film thickness, J&W Scientific, USA) and a (DVB/CAR/PDMS) 50/30 μm extraction head (J&W Scientific, Santa Clara, CA, USA) was used in conjunction with an Agilent triple quadrupole mass selective detector. Quantitative analysis was performed using similar compounds as references [6]. The mass spectrometry conditions were as follows: EI ionization source, ion source temperature 230 °C, quadrupole temperature 150 °C, ionization voltage 70 eV, mass scan range 30~500 m/z, transfer line temperature 280 °C. Data report collection was conducted in full scan mode.

First, determine the LOQ (0.049 mg/L) of volatile organic compounds in loquat beer using the method by Piergiovanni [43], so that the measured volatile organic compounds in all samples can be accurately quantified. Then, randomly test several QC samples, and after the instrument signal is stabilized, the detection of loquat beer will begin. QC sample will be detected between the 2 sample types to examine the instrument’s stability. The retention times of the normal alkane standard will be determined using the same method, and this will be used to calculate the Retention Index (RI) of each volatile organic compound in the samples.

Ethyl hexanoate was used for quantifying esters, 2-octanol for alcohols, n-hexane for alkanes, cyclohexanone for ketones, benzaldehyde for aldehydes, and phenol for aromatics. These standard compounds were purchased from Sigma-Aldrich (Shanghai, China). The six standard compounds were mixed with anhydrous ethanol, and different volumes of the alcohol solution containing the six standard compounds were added to pure water with a pH of 3.5 and an ethanol content of 4.0% (this solution was prepared to simulate the beer matrix) to prepare water solutions containing different concentrations of the mixed standard compounds. By establishing a regression equation between the peak area and concentration of the standard compounds, the peak areas of the VOCs in the samples were quantitatively analyzed using the equation.

The odor thresholds of the VOCs in water and the OAVs were calculated based on the latest reported values [44].

The calculation formula for OAV is OAV = Ci/Ti, where Ci represents the concentration of the compound in the loquat beer sample, and Ti represents the odor threshold of the compound in water. When OAV ≥ 1, it indicates that the compound contributes significantly to the aroma of the loquat beer [45].

### 4.6. Data Analysis

The results were analyzed using SPSS Statistics 26 to assess significant differences and perform variance analysis between the data. Spider web plots were generated using Origin 2020 software. Principal component analysis plots were created using SMICA 13.0 software.

## 5. Conclusions

In this study, sensory analysis, physicochemical analysis, and VOCs analysis were conducted on loquat beer brewed with different proportions of loquat juice. The results showed that during the preparation of loquat beer, adding an appropriate amount of loquat juice (40%) and mixing it with beer base materials (water, malt, hops, yeast), followed by fermentation, clarification, filtration, and other production processes, resulted in loquat beer with a unique flavor, soft texture, and overall good sensory quality. As the proportion of loquat juice increased, the organic acids in the beer exhibited different trends. VOCs analysis revealed that esters were the main VOCs, and 23 key VOCs were identified through correlation analysis and VIP value screening, providing a scientific basis for the development of high-quality loquat beer.

## Figures and Tables

**Table 1:** Physicochemical indexes of loquat beer.

	PP-0	PP-20	PP-40	PP-60	PP-80	PP-100	*p*
pH	4.14 ± 0.09 ^a^	4.12 ± 0.07 ^a^	3.88 ± 0.11 ^b^	3.56 ± 0.06 ^c^	3.53 ± 0.08 ^cd^	3.51 ± 0.06 ^d^	*
Alcohol (% vol)	4.85 ± 0.12 ^a^	4.55 ± 0.14 ^b^	4.20 ± 0.09 ^c^	3.85 ± 0.10 ^d^	3.81 ± 0.11 ^d^	3.68 ± 0.10 ^e^	*
Density (kg/m^3^)	1024.20 ± 1.26 ^a^	1015.14 ± 1.12 ^b^	1016.50 ± 1.08 ^b^	1012.10 ± 1.15 ^c^	1008.11 ± 1.20 ^d^	1006.12 ± 1.14 ^d^	*
Specific gravity (°P)	1030.12 ± 1.27 ^a^	1021.23 ± 1.14 ^b^	1022.10 ± 1.13 ^b^	1014.40 ± 1.18 ^c^	1011.30 ± 1.22 ^cd^	1010.06 ± 1.17 ^d^	*
Succinic acid (mg/L)	17.23 ± 0.42 ^c^	29.54 ± 0.62 ^b^	52.74 ± 1.01 ^a^	51.78 ± 1.20 ^a^	30.45 ± 1.06 ^b^	31.57 ± 0.91 ^b^	*
Malic acid (mg/L)	154.64 ± 2.24 ^f^	183.34 ± 3.24 ^e^	213.78 ± 3.42 ^d^	254.20 ± 3.25 ^c^	283.40 ± 3.33 ^b^	330.37 ± 3.15 ^a^	**
Lactic acid (mg/L)	175.40 ± 2.52 ^f^	395.60 ± 3.25 ^b^	480.42 ± 2.12 ^a^	360.56 ± 3.41 ^c^	335.50 ± 4.62 ^d^	273.58 ± 3.01 ^e^	**
Acetic acid (mg/L)	123.40 ± 3.80 ^a^	95.56 ± 2.06 ^b^	93.00 ± 1.52 ^b^	70.47 ± 2.57 ^c^	52.40 ± 0.68 ^d^	50.57 ± 1.03 ^d^	*
Tartaric acid (mg/L)	24.46 ± 0.90 ^e^	44.50 ± 1.21 ^d^	75.67 ± 1.48 ^c^	90.48 ± 1.70 ^b^	109.57 ± 2.06 ^a^	111.78 ± 1.59 ^a^	*
Citric acid (mg/L)	229.54 ± 2.67 ^d^	231.76 ± 4.82 ^d^	268.86 ± 2.90 ^c^	297.57 ± 3.30 ^b^	338.75 ± 3.70 ^a^	340.78 ± 3.13 ^a^	*

According to Tukey’s test, there was no significant difference in the data representation of the same letter in the same row. The symbol * was compared with PP-0: ** *p* < 0.01; * *p* < 0.05.

**Table 2:** Concentration of VOCs in loquat beer (mg/L).

CAS	Compounds	PP-0	PP-20	PP-40	PP-60	PP-80	PP-100
141–78-6	Ethyl acetate	16.65 ± 1.12 ^c^	26.05 ± 1.54 ^b^	47.51 ± 1.88 ^a^	28.51 ± 1.11 ^b^	27.72 ± 1.23 ^b^	47.57 ± 1.15 ^a^
106-32-1	Ethyl caprylate	18.48 ± 1.11 ^b^	42.54 ± 2.32 ^a^	9.74 ± 1.44 ^d^	15.85 ± 1.41 ^c^	4.41 ± 0.78 ^e^	4.69 ± 0.66 ^e^
103-45-7	Phenethyl acetate	13.92 ± 1.52 ^d^	47.90 ± 2.62 ^b^	223.41 ± 5.02 ^a^	14.05 ± 1.91 ^d^	42.01 ± 2.10 ^c^	13.92 ± 1.72 ^d^
110-38-3	Ethyl caprate	10.33 ± 0.32 ^b^	27.02 ± 1.57 ^a^	3.90 ± 0.11 ^d^	4.85 ± 0.42 ^c^	2.51 ± 0.09 ^e^	1.57 ± 0.13 ^f^
123-92-2	Isoamyl acetate	8.18 ± 0.31 ^cd^	15.69 ± 0.32 ^b^	35.11 ± 0.15 ^a^	7.53 ± 0.56 ^d^	9.66 ± 0.15 ^c^	5.46 ± 0.13 ^e^
104-61-0	γ-Nonanolactone	7.73 ± 0.42 ^b^	8.09 ± 0.13 ^a^	1.31 ± 0.17 ^d^	4.33 ± 0.12 ^c^	1.77 ± 0.39 ^d^	0.24 ± 0.06 ^e^
123-66-0	Ethyl hexanoate	8.67 ± 0.32 ^a^	7.47 ± 0.45 ^b^	4.49 ± 0.16 ^c^	7.48 ± 0.20 ^b^	3.38 ± 0.12 ^c^	2.81 ± 0.06 ^d^
84-69-5	Diisobutyl phthalate	5.32 ± 0.35 ^d^	11.71 ± 0.62 ^b^	7.81 ± 0.54 ^c^	14.26 ± 1.42 ^a^	8.06 ± 0.92 ^c^	10.07 ± 1.52 ^b^
1962-75-0	Dibutyl terephthalate	3.30 ± 0.12 ^bc^	3.08 ± 0.15 ^c^	3.58 ± 0.30 ^b^	5.93 ± 0.62 ^a^	3.38 ± 0.30 ^b^	3.10 ± 0.14 ^c^
624-41-9	2-Methylbutyl acetate	3.36 ± 0.16 ^c^	5.27 ± 0.22 ^b^	8.23 ± 0.54 ^a^	2.55 ± 0.14 ^de^	2.85 ± 0.16 ^d^	2.30 ± 0.11 ^e^
119-36-8	Methyl salicylate	0.10 ± 0.02 ^e^	1.52 ± 0.04 ^b^	0.21 ± 0.01 ^d^	13.24 ± 0.04 ^a^	0.28 ± 0.01 ^d^	0.56 ± 0.02 ^c^
106-33-2	Ethyl laurate	2.95 ± 0.13 ^b^	10.24 ± 0.16 ^a^	0.69 ± 0.07 ^d^	0.96 ± 0.07 ^c^	0.78 ± 0.04 ^d^	0.18 ± 0.03 ^e^
93-89-0	Ethyl benzoate	0.66 ± 0.03 ^d^	2.16 ± 0.16 ^c^	6.69 ± 0.39 ^b^	9.64 ± 1.15 ^a^	2.15 ± 0.47 ^c^	5.87 ± 0.99 ^b^
2021-28-5	3-Phenylpropionate ethyl	0.51 ± 0.04 ^de^	7.29 ± 1.02 ^a^	0.62 ± 0.09 ^c^	1.50 ± 0.04 ^b^	0.45 ± 0.03 ^e^	0.15 ± 0.02 ^f^
77-68-9	3-Hydroxy-2,2,4-trimethylpentyl isobutyrate	1.33 ± 0.03 ^b^	1.12 ± 0.04 ^d^	1.24 ± 0.05 ^cd^	2.59 ± 0.52 ^a^	1.29 ± 0.05 ^b^	1.38 ± 0.63 ^b^
101-97-3	Ethyl phenylacetate	0.52 ± 0.01 ^d^	1.62 ± 0.08 ^a^	1.04 ± 0.12 ^c^	1.27 ± 0.09 ^b^	0.41 ± 0.04 ^e^	0.54 ± 0.06 ^d^
105-37-3	Ethyl propionate	0.56 ± 0.02 ^a^	0.23 ± 0.02 ^d^	0.25 ± 0.01 ^d^	0.45 ± 0.02 ^b^	0.19 ± 0.02 ^d^	0.35 ± 0.01 ^c^
110-19-0	Isobutyl acetate	0.91 ± 0.07 ^b^	1.14 ± 0.08 ^b^	2.39 ± 0.95 ^a^	1.04 ± 0.73 ^b^	0.57 ± 0.04 ^c^	0.48 ± 0.06 ^c^
868-57-5	2-Methylbutyrate methyl	0.39 ± 0.03 ^b^	0.52 ± 0.02 ^a^	ND	ND	ND	ND
97-62-1	Ethyl isobutyrate	0.25 ± 0.02 ^b^	0.19 ± 0.02 ^c^	0.40 ± 0.02 ^a^	0.27 ± 0.01 ^b^	0.13 ± 0.01 ^d^	0.14 ± 0.02 ^d^
105-54-4	Ethyl butyrate	0.79 ± 0.04 ^a^	0.56 ± 0.02 ^b^	0.53 ± 0.01 ^b^	0.70 ± 0.05 ^a^	0.38 ± 0.02 ^c^	0.33 ± 0.01 ^c^
7452-79-1	2-Methylbutyrate ethyl	0.09 ± 0.01 ^e^	0.25 ± 0.01 ^d^	0.43 ± 0.03 ^c^	0.79 ± 0.05 ^a^	0.50 ± 0.03 ^b^	0.57 ± 0.04 ^b^
590-01-2	Butyl propionate	0.21 ± 0.01 ^b^	0.21 ± 0.02 ^b^	0.09 ± 0.03 ^c^	0.32 ± 0.02 ^a^	0.05 ± 0.01 ^c^	0.32 ± 0.01 ^a^
106-70-7	Methyl hexanoate	ND	ND	0.05 ± 0.01 ^b^	0.06 ± 0.01 ^b^	ND	0.09 ± 0.01 ^a^
93-58-3	Methyl benzoate	0.12 ± 0.01 ^d^	0.39 ± 0.02 ^a^	0.40 ± 0.01 ^a^	0.25 ± 0.01 ^c^	0.15 ± 0.01 ^d^	0.29 ± 0.02 ^b^
106-30-9	Ethyl heptanoate	0.58 ± 0.02 ^a^	0.53 ± 0.03 ^a^	0.15 ± 0.01 ^c^	0.23 ± 0.01 ^b^	0.07 ± 0.01 ^d^	0.06 ± 0.01 ^d^
111-11-5	Caprylic acid methyl ester	0.07 ± 0.00 ^c^	0.17 ± 0.02 ^b^	0.17 ± 0.02 ^b^	0.25 ± 0.03 ^a^	ND	0.15 ± 0.01 ^b^
123-25-1	Diethyl succinate	0.08 ± 0.01 ^c^	ND	ND	0.18 ± 0.03 ^b^	1.58 ± 0.06 ^a^	0.07 ± 0.02 ^c^
118-61-6	2-Hydroxybenzoate ethyl	0.01 ± 0.00 ^e^	0.47 ± 0.02 ^a^	0.12 ± 0.01 ^b^	0.42 ± 0.03 ^a^	0.07 ± 0.02 ^d^	0.09 ± 0.01 ^cd^
123-29-5	Ethyl nonanoate	0.41 ± 0.02 ^b^	0.96 ± 0.05 ^a^	0.20 ± 0.01 ^c^	0.40 ± 0.03 ^b^	0.13 ± 0.01 ^d^	0.13 ± 0.02 ^d^
110-42-9	Methyl decanoate	0.12 ± 0.01 ^b^	0.16 ± 0.02 ^a^	0.05 ± 0.01 ^c^	0.06 ± 0.01 ^c^	ND	ND
2035-99-6	Caprylic acid isoamyl ester	0.12 ± 0.01 ^c^	1.21 ± 0.04 ^a^	0.13 ± 0.02 ^c^	0.36 ± 0.04 ^b^	0.07 ± 0.03 ^d^	ND
103-36-6	Ethyl cinnamate	0.13 ± 0.01 ^e^	0.27 ± 0.02 ^d^	0.69 ± 0.04 ^a^	0.58 ± 0.04 ^b^	0.23 ± 0.03 ^d^	0.49 ± 0.03 ^c^
706-14-9	γ-Decalactone	0.29 ± 0.01 ^a^	0.31 ± 0.02 ^a^	0.24 ± 0.01 ^b^	0.27 ± 0.03 ^ab^	0.16 ± 0.01 ^c^	0.33 ± 0.03 ^a^
628-97-7	Palmitic acid ethyl ester	0.84 ± 0.06 ^b^	0.84 ± 0.07 ^b^	0.21 ± 0.03 ^d^	1.16 ± 0.13 ^a^	0.63 ± 0.04 ^c^	0.09 ± 0.01 ^d^
	Σ Esters	108.00	227.18	362.11	142.33	116.02	104.42
60-12-8	Phenylethyl alcohol	454.51 ± 4.56 ^c^	606.63 ± 7.93 ^a^	577.95 ± 9.53 ^b^	621.97 ± 10.97 ^a^	324.25 ± 5.46 ^d^	37.29 ± 3.54 ^e^
123-51-3	3-Methyl-1-butanol	104.22 ± 5.33 ^c^	82.52 ± 2.32 ^e^	220.21 ± 6.32 ^a^	115.12 ± 4.22 ^b^	90.65 ± 4.18 ^d^	35.96 ± 2.23 ^f^
137-32-6	2-Methyl-1-butanol	60.68 ± 3.16 ^b^	44.29 ± 2.75 ^c^	90.16 ± 3.86 ^a^	61.66 ± 2.72 ^b^	43.67 ± 3.45 ^c^	32.35 ± `1.78 ^d^
78-83-1	2-Methyl-1-propanol	14.78 ± 1.01 ^d^	40.12 ± 1.36 ^a^	34.02 ± 1.53 ^b^	23.98 ± 1.08 ^c^	12.41 ± 0.36 ^d^	25.48 ± 0.27 ^c^
143-08-8	1-Nonanol	8.12 ± 0.96 ^b^	6.81 ± 0.74 ^c^	5.25 ± 0.36 ^d^	12.95 ± 0.86 ^a^	3.76 ± 0.42 ^e^	5.96 ± 0.80 ^d^
106-24-1	Geraniol	3.60 ± 0.26 ^b^	4.43 ± 0.18 ^a^	1.89 ± 0.38 ^c^	3.91 ± 0.63 ^b^	1.11 ± 0.39 ^d^	1.30 ± 0.27 ^d^
111-27-3	1-Hexanol	1.58 ± 0.10 ^c^	1.83 ± 0.17 ^b^	1.07 ± 0.34 ^d^	4.17 ± 0.27 ^a^	0.56 ± 0.08 ^e^	4.20 ± 0.24 ^a^
513-85-9	2,3-Butanediol	1.62 ± 0.20 ^c^	0.45 ± 0.03 ^d^	0.48 ± 0.02 ^d^	2.07 ± 0.07 ^b^	0.04 ± 0.00 ^e^	4.14 ± 0.16 ^a^
78-70-6	Linalool	1.22 ± 0.02 ^d^	2.35 ± 0.03 ^b^	2.85 ± 0.06 ^a^	1.47 ± 0.03 ^c^	1.46 ± 0.02 ^c^	0.84 ± 0.04 ^e^
628-99-9	2-Nonanol	1.39 ± 0.13 ^c^	3.00 ± 0.20 ^a^	0.58 ± 0.06 ^d^	1.77 ± 0.09 ^b^	0.14 ± 0.02 ^e^	0.62 ± 0.02 ^d^
106-22-9	Citronellol	1.57 ± 0.02 ^b^	1.85 ± 0.07 ^a^	1.28 ± 0.11 ^c^	1.97 ± 0.12 ^a^	0.66 ± 0.05 ^e^	0.91 ± 0.04 ^d^
112-53-8	1-Dodecanol	1.95 ± 0.03 ^b^	1.66 ± 0.07 ^c^	1.96 ± 0.08 ^b^	2.61 ± 0.10 ^a^	0.83 ± 0.05 ^e^	1.53 ± 0.10 ^d^
112-30-1	1-Decanol	0.79 ± 0.02 ^c^	1.87 ± 0.14 ^a^	0.83 ± 0.07 ^c^	1.30 ± 0.12 ^b^	0.31 ± 0.05 ^d^	0.37 ± 0.04 ^d^
71-36-3	1-Butanol	0.08 ± 0.02 ^c^	0.13 ± 0.03 ^b^	0.16 ± 0.02 ^b^	0.09 ± 0.01 ^c^	ND	0.25 ± 0.00 ^a^
626-89-1	4-Methyl-1-pentanol	0.16 ± 0.03 ^a^	0.07 ± 0.01 ^b^	ND	0.12 ± 0.02 ^a^	0.06 ± 0.01 ^b^	ND
589-35-5	3-Methyl-1-pentanol	0.08 ± 0.01 ^c^	0.12 ± 0.02 ^b^	0.10 ± 0.01 ^c^	0.18 ± 0.03 ^a^	0.10 ± 0.02 ^c^	0.16 ± 0.03 ^a^
544-12-7	Trans-3-hexen-1-ol	0.39 ± 0.06 ^c^	0.26 ± 0.06 ^d^	0.49 ± 0.06 ^b^	0.31 ± 0.06 ^d^	0.75 ± 0.06 ^a^	0.64 ± 0.06 ^a^
126-30-7	Neopentyl glycol	0.05 ± 0.01 ^e^	0.17 ± 0.06 ^d^	0.34 ± 0.09 ^c^	0.80 ± 0.04 ^b^	0.15 ± 0.03 ^d^	1.43 ± 0.07 ^a^
111-70-6	1-Heptanol	0.72 ± 0.05 ^a^	0.63 ± 0.06 ^b^	0.40 ± 0.02 ^c^	0.65 ± 0.04 ^b^	0.20 ± 0.02 ^c^	0.60 ± 0.03 ^b^
3391-86-4	1-Octen-3-ol	0.51 ± 0.03 ^b^	3.18 ± 0.09 ^a^	0.37 ± 0.03 ^c^	0.49 ± 0.05 ^b^	0.14 ± 0.01 ^d^	0.16 ± 0.01 ^d^
505-10-2	3-Methylthiopropanol	0.09 ± 0.01 ^c^	0.23 ± 0.05 ^b^	0.34 ± 0.04 ^a^	0.08 ± 0.01 ^c^	0.06 ± 0.00 ^d^	ND
104-76-7	2-Ethylhexanol	0.77 ± 0.05 ^b^	0.61 ± 0.07 ^c^	0.77 ± 0.03 ^b^	1.40 ± 0.10 ^a^	0.32 ± 0.09 ^d^	1.29 ± 0.11 ^a^
100-51-6	Benzyl alcohol	0.16 ± 0.02 ^c^	0.26 ± 0.02 ^b^	0.11 ± 0.01 ^d^	0.69 ± 0.03 ^a^	0.11 ± 0.02 ^d^	0.07 ± 0.01 ^e^
18409-17-1	(E)-2-Octen-1-ol	0.18 ± 0.02 ^b^	0.20 ± 0.04 ^b^	0.20 ± 0.02 ^b^	0.37 ± 0.02 ^a^	0.14 ± 0.01 ^c^	0.11 ± 0.01 ^d^
10340-23-5	(Z)-3-Nonen-1-ol	0.16 ± 0.03 ^e^	0.44 ± 0.01 ^c^	0.32 ± 0.01 ^d^	1.76 ± 0.03 ^a^	0.14 ± 0.02 ^e^	0.80 ± 0.05 ^b^
31502-14-4	(E)-2-Nonen-1-ol	0.32 ± 0.02 ^b^	0.34 ± 0.03 ^b^	0.35 ± 0.03 ^b^	0.71 ± 0.04 ^a^	0.24 ± 0.01 ^c^	0.16 ± 0.02 ^d^
562-74-3	Terpinen-4-ol	0.10 ± 0.01 ^c^	0.15 ± 0.01 ^b^	0.24 ± 0.02 ^a^	0.13 ± 0.02 ^bc^	0.11 ± 0.01 ^c^	0.06 ± 0.01 ^d^
98-55-5	α-Terpineol	0.20 ± 0.01 ^c^	0.30 ± 0.02 ^a^	0.21 ± 0.03 ^bc^	0.30 ± 0.06 ^a^	0.24 ± 0.02 ^b^	0.34 ± 0.05 ^a^
40716-66-3	Trans-(E)-nerolidol	0.56 ± 0.03 ^a^	0.48 ± 0.02 ^b^	0.17 ± 0.02 ^d^	0.48 ± 0.02 ^b^	0.18 ± 0.02 ^d^	0.25 ± 0.03 ^c^
77-53-2	Cedrol	0.34 ± 0.02 ^d^	0.64 ± 0.03 ^b^	0.39 ± 0.02 ^c^	0.92 ± 0.07 ^a^	0.30 ± 0.02 ^d^	0.66 ± 0.08 ^b^
106-28-5	(E,E)-Farnesol	0.29 ± 0.02 ^c^	0.42 ± 0.03 ^b^	0.09 ± 0.01 ^e^	0.96 ± 0.05 ^a^	0.14 ± 0.01 ^d^	0.06 ± 0.01 ^f^
	Σ Alcohols	661.21	806.45	943.59	865.43	483.25	157.98
543-59-9	1-Chloro-pentane	105.06 ± 4.36 ^c^	82.76 ± 3.64 ^e^	222.10 ± 4.98 ^a^	115.56 ± 2.64 ^b^	91.24 ± 3.86 ^d^	51.09 ± 2.64 ^f^
16746-87-5	2,4-Dimethyl-1-hexene	46.35 ± 2.43 ^b^	33.58 ± 1.75 ^c^	62.49 ± 2.53 ^a^	48.14 ± 3.20 ^b^	32.50 ± 1.24 ^c^	26.23 ± 3.26 ^d^
108-88-3	Toluene	5.48 ± 0.86 ^c^	2.44 ± 0.24 ^e^	4.27 ± 0.20 ^d^	6.38 ± 0.30 ^b^	1.37 ± 0.28 ^f^	8.01 ± 0.69 ^a^
91-20-3	Naphthalene	1.09 ± 0.12 ^a^	0.64 ± 0.06 ^b^	0.61 ± 0.04 ^b^	1.01 ± 0.08 ^a^	0.43 ± 0.03 ^d^	0.55 ± 0.03 ^c^
50894-66-1	(+)-α-Funebrene	0.20 ± 0.02 ^b^	0.18 ± 0.01 ^b^	0.13 ± 0.02 ^c^	0.26 ± 0.03 ^a^	0.12 ± 0.02 ^c^	0.25 ± 0.02 ^a^
100-42-5	Styrene	0.44 ± 0.03 ^c^	0.37 ± 0.04 ^cd^	0.34 ± 0.03 ^d^	0.55 ± 0.02 ^b^	0.16 ± 0.02 ^e^	0.71 ± 0.03 ^a^
106-42-3	p-Xylene	0.16 ± 0.01 ^b^	0.12 ± 0.02 ^c^	0.15 ± 0.01 ^b^	0.17 ± 0.01 ^b^	0.08 ± 0.02 ^d^	0.30 ± 0.02 ^a^
123-35-3	Myrcene	0.21 ± 0.02 ^b^	0.20 ± 0.02 ^bc^	0.18 ± 0.01 ^c^	0.32 ± 0.03 ^a^	0.13 ± 0.02 ^d^	0.22 ± 0.01 ^b^
3779-61-1	(E)-β-Ocimene	0.09 ± 0.01 ^a^	0.10 ± 0.01 ^a^	0.06 ± 0.00 ^b^	0.11 ± 0.01 ^a^	0.05 ± 0.00 ^b^	0.06 ± 0.00 ^b^
	Σ Alkanes	159.08	120.38	290.34	172.51	126.08	87.42
112-31-2	Decanal	6.73 ± 0.34 ^b^	5.98 ± 0.74 ^c^	4.79 ± 0.30 ^d^	10.78 ± 0.90 ^a^	4.06 ± 0.23 ^e^	3.25 ± 0.55 ^f^
124-19-6	Nonanal	3.52 ± 0.10 ^c^	3.21 ± 0.12 ^d^	4.73 ± 0.21 ^b^	5.86 ± 0.22 ^a^	2.62 ± 0.17 ^e^	1.40 ± 0.10 ^f^
122-78-1	Benzeneacetaldehyde	1.79 ± 0.16 ^a^	0.95 ± 0.09 ^b^	0.72 ± 0.04 ^c^	1.62 ± 0.10 ^a^	0.68 ± 0.07 ^c^	0.24 ± 0.05 ^d^
432-25-7	β-Cyclocitral	0.37 ± 0.03 ^b^	1.49 ± 0.04 ^a^	0.21 ± 0.01 ^c^	0.38 ± 0.02 ^b^	0.14 ± 0.01 ^d^	0.35 ± 0.03 ^b^
3913-81-3	(E)-3-Heptylacrolein	0.15 ± 0.01 ^a^	0.09 ± 0.01 ^b^	0.06 ± 0.01 ^c^	0.09 ± 0.01 ^b^	ND	ND
112-44-7	Undecanal	0.30 ± 0.01 ^b^	0.24 ± 0.01 ^c^	0.16 ± 0.01 ^e^	0.36 ± 0.02 ^a^	0.19 ± 0.01 ^d^	0.13 ± 0.01 ^f^
104-67-6	Undecan-4-olide	0.12 ± 0.01 ^b^	0.12 ± 0.01 ^b^	0.10 ± 0.01 ^b^	0.17 ± 0.02 ^a^	0.11 ± 0.02 ^b^	0.17 ± 0.02 ^a^
1620-98-0	3,5-Di-tert-butyl-4-hydroxybenzaldehyde	0.21 ± 0.03 ^d^	0.38 ± 0.04 ^b^	0.39 ± 0.02 ^b^	0.61 ± 0.02 ^a^	0.32 ± 0.01 ^c^	0.57 ± 0.02 ^a^
111-71-7	Heptanal	0.17 ± 0.01 ^b^	0.11 ± 0.01 ^c^	0.08 ± 0.02 ^d^	0.20 ± 0.02 ^a^	0.10 ± 0.01 ^c^	0.08 ± 0.01 ^d^
100-52-7	Benzaldehyde	0.39 ± 0.02 ^a^	0.14 ± 0.01 ^d^	0.13 ± 0.02 ^d^	0.27 ± 0.02 ^b^	0.24 ± 0.02 ^c^	0.19 ± 0.03 ^c^
	Σ Aldehyde	13.75	12.72	11.38	20.34	8.44	6.39
689-67-8	6,10-Dimethyl-5,9-undecadien-2-one	1.26 ± 0.08 ^e^	1.64 ± 0.07 ^d^	5.34 ± 0.14 ^a^	4.21 ± 0.18 ^b^	0.86 ± 0.06 ^f^	2.98 ± 0.10 ^c^
5704-20-1	2-Hydroxy-3-pentanone	0.85 ± 0.04 ^e^	1.01 ± 0.08 ^d^	0.65 ± 0.03 ^f^	1.28 ± 0.06 ^c^	5.80 ± 0.14 ^a^	2.43 ± 0.10 ^b^
112-12-9	2-Undecanone	0.09 ± 0.01 ^c^	0.14 ± 0.02 ^b^	ND	0.21 ± 0.04 ^a^	ND	ND
821-55-6	2-Nonanone	0.23 ± 0.03 ^b^	0.35 ± 0.04 ^a^	0.08 ± 0.01 ^c^	0.32 ± 0.03 ^a^	ND	0.06 ± 0.01 ^c^
110-93-0	6-Methyl-5-hepten-2-one	0.27 ± 0.04 ^b^	0.09 ± 0.02 ^d^	0.25 ± 0.03 ^bc^	0.23 ± 0.02 ^c^	0.12 ± 0.01 ^d^	0.43 ± 0.03 ^a^
4312-99-6	1-Octen-3-one	0.10 ± 0.01 ^d^	0.08 ± 0.01 ^b^	0.09 ± 0.02 ^b^	0.20 ± 0.01 ^a^	0.05 ± 0.01 ^c^	ND
79-77-6	β-Lonone	0.06 ± 0.01 ^e^	0.30 ± 0.03 ^d^	0.83 ± 0.04 ^a^	0.77 ± 0.03 ^a^	0.39 ± 0.02 ^c^	0.67 ± 0.01 ^b^
	Σ Ketones	2.86	3.61	7.24	7.23	7.22	6.57
334-48-5	Decanoic acid	11.25 ± 0.86 ^b^	16.78 ± 0.67 ^a^	6.51 ± 0.20 ^d^	8.30 ± 0.68 ^c^	3.54 ± 0.20 ^f^	4.43 ± 0.10 ^e^
503-74-2	Isovaleric acid	1.84 ± 0.11 ^c^	2.25 ± 0.12 ^b^	8.95 ± 0.16 ^a^	1.44 ± 0.20 ^d^	1.95 ± 0.16 ^c^	2.15 ± 0.21 ^b^
111-14-8	Heptanoic acid	1.94 ± 0.10 ^a^	1.33 ± 0.14 ^b^	0.66 ± 0.03 ^d^	1.10 ± 0.04 ^c^	0.26 ± 0.02 ^f^	0.40 ± 0.02 ^e^
112-05-0	Nonanoic acid	1.81 ± 0.14 ^e^	2.36 ± 0.16 ^d^	2.59 ± 0.07 ^c^	2.97 ± 0.19 ^b^	1.38 ± 0.10 ^f^	3.21 ± 0.21 ^a^
	Σ Organic acids	16.84	22.71	18.71	13.81	7.13	10.18
5875-45-6	2,5-Di-Tert-butylphenol	82.02 ± 2.06 ^d^	99.92 ± 2.12 ^c^	97.42 ± 3.01 ^c^	171.57 ± 4.14 ^a^	60.30 ± 1.46 ^e^	133.01 ± 3.64 ^b^
128-39-2	2,6-Di-tert-butylphenol	1.37 ± 0.12 ^ab^	0.99 ± 0.10 ^c^	1.25 ± 0.15 ^b^	1.48 ± 0.13 ^a^	0.64 ± 0.05 ^d^	1.01 ± 0.06 ^c^
7786-61-0	4-Hydroxy-3-methoxystyrene	0.29 ± 0.01 ^b^	0.37 ± 0.02 ^a^	0.07 ± 0.01 ^d^	0.15 ± 0.01 ^c^	0.06 ± 0.01 ^d^	0.12 ± 0.02 ^c^
97-53-0	Eugenol	ND	0.08 ± 0.01 ^b^	ND	0.13 ± 0.02 ^a^	ND	0.06 ± 0.01 ^bc^
	Σ Phenols	83.69	101.36	98.73	173.32	61.00	134.2
	Σ All	1045.43	1294.41	1732.10	1394.97	809.14	507.16

Note: ND indicates a content below the limit of quantification (LOQ). According to Tukey’s test, there was no significant difference in the data representation of the same letter in the same row.

**Table 3:** OAVs of VOCs in loquat beer.

Compounds	Odor Threshold mg/L	PP-0	PP-20	PP-40	PP-60	PP-80	PP-100	Odor Description
Ethyl acetate	0.005	3331.1 ± 224.2 ^c^	5211.4 ± 308.6 ^b^	9500.7 ± 376.7 ^a^	5701.5 ± 222.1 ^b^	5544.3 ± 246.0 ^b^	9513.7 ± 230.4 ^a^	Fruity aroma
Ethyl caprylate	1.9 × 10^−2^	957.6 ± 58.6 ^b^	2204.4 ± 122.3 ^a^	504.8 ± 76.4 ^d^	821.3 ± 74.7 ^c^	228.7 ± 41.5 ^e^	243.4 ± 35.0 ^e^	Brandy fragrance
Phenethyl acetate	0.250	56.2 ± 6.4 ^d^	192.4 ± 10.6 ^b^	894.8 ± 20.1 ^a^	55.7 ± 8.6 ^d^	167.9 ± 8.6 ^c^	56.0 ± 7.4 ^d^	Sweet fragrance
Ethyl caprate	0.005	2066.4 ± 64.4 ^b^	5404.3 ± 314.5 ^a^	779.1 ± 22.4 ^d^	969.0 ± 84.5 ^c^	502.5 ± 18.0 ^e^	314.7 ± 26.0 ^f^	Coconut fragrance
Isoamyl acetate	1.5 × 10^−4^	54,520.1 ± 2067.5 ^cd^	104,583.1 ± 2133.5 ^b^	234,046.3 ± 1000.3 ^a^	50,208.2 ± 3733.0 ^d^	64,396.1 ± 1000.5 ^c^	36,395.4 ± 867.4 ^e^	Banana fragrance
γ-Nonanolactone	9.7×10^−3^	797.5 ± 43.4 ^b^	834.8 ± 13.4 ^a^	135.2 ± 18.3 ^d^	446.1 ± 12.1 ^c^	183.0 ± 40.2 ^d^	25.1 ± 6.2 ^e^	Coconut aroma, fennel
Ethyl hexanoate	0.005	1734.1 ± 64.9 ^a^	1494.3 ± 90.3 ^b^	898.4 ± 32.3 ^c^	1496.8 ± 40.0 ^b^	675.8 ± 24.5 ^c^	562.0 ± 12.5 ^d^	Fruity aroma
2-Methylbutyl acetate	0.005	673.3 ± 32.4 ^c^	1055.4 ± 44.3 ^b^	1644.7 ± 108.8 ^a^	509.0 ± 28.9 ^de^	569.1 ± 32.4 ^d^	461.2 ± 22.1 ^e^	Sweet and fruity aroma
Methyl salicylate	0.04	3.1 ± 0.5 ^e^	38.2 ± 1 ^b^	5.2 ± 0.3 ^e^	331.1 ± 1 ^a^	7.0 ± 0.3 ^d^	14.2 ± 0.5 ^c^	Fragrance of holly leaves
Ethyl benzoate	0.055	12.6 ± 0.5 ^d^	39.2 ± 2.9 ^c^	120.1 ± 7.4 ^b^	174.2 ± 21.3 ^a^	39.0 ± 9.0 ^c^	106.2 ± 18.6 ^b^	Holly oil fragrance
Ethyl phenylacetate	0.155	3.2 ± 0.1 ^d^	10.1 ± 0.5 ^a^	6.8 ± 0.8 ^c^	7.8 ± 0.6 ^b^	3.0 ± 0.3 ^d^	3.0 ± 0.4 ^d^	Honey aroma
Ethyl propionate	0.01	56.1 ± 2.5 ^a^	23.2 ± 2.2 ^d^	25.4 ± 1.1 ^d^	45.1 ± 2.7 ^b^	18.7 ± 2.6 ^d^	35.4 ± 1.2 ^c^	Pineapple aroma
Isobutyl acetate	0.025	36.2 ± 3.2 ^b^	46.4 ± 3.5 ^b^	95.6 ± 4.4 ^a^	42.4 ± 3.2 ^b^	23.0 ± 2.2 ^c^	19.1 ± 2.9 ^c^	Fruity aroma
2-Methylbutyrate methyl	2.5 × 10^−4^	1558.2 ± 120.2 ^b^	2066.1 ± 80.8 ^a^	89.4 ± 40.4 ^d^	169.0 ± 40.8 ^cd^	85.7 ± 40.3 ^d^	196.6 ± 40.2 ^c^	Apple and rum aromas
Ethyl isobutyrate	2 × 10^−5^	12,732.1 ± 1000.5 ^b^	9271.2 ± 1000.2 ^c^	20,065.3 ± 1000.8 ^a^	13,565.8 ± 500.7 ^b^	6747.9 ± 500.9 ^d^	6971.2 ± 1000.6 ^d^	Fruit and cream fragrance
Ethyl butyrate	9 × 10^−4^	877.1 ± 44.4 ^a^	626.3 ± 22.3 ^b^	593.4 ± 11.2 ^b^	774.7 ± 56.9 ^a^	423.9 ± 22 ^c^	366.0 ± 11 ^c^	Pineapple aroma
2-Methylbutyrate ethyl	1.3 × 10^−5^	7094.2 ± 769.4 ^e^	19,443.8 ± 769.3 ^d^	32,693.1 ± 2308.2 ^c^	60,679.2 ± 3846.9 ^a^	38,085.3 ± 3077.9 ^b^	43,745.4 ± 4615.3 ^b^	Fruit peel fragrance
Methyl benzoate	0.073	2.1 ± 0.1 ^d^	5.0 ± 0.3 ^a^	5.1 ± 0.1 ^a^	3.1 ± 0.1 ^c^	2.2 ± 0.1 ^d^	4.2 ± 0.3 ^b^	Floral and cherry fragrance
Ethyl heptanoate	1.9 × 10^−3^	303.1 ± 11.4 ^a^	282.3 ± 16.9 ^a^	78.3 ± 5.5 ^c^	121.7 ± 5.2 ^b^	37.3 ± 5.1 ^d^	34.0 ± 5.2 ^d^	Pineapple fragrance
Methyl decanoate	4.3 × 10^−3^	28.0 ± 2.1 ^b^	36.1 ± 5.3 ^a^	12.2 ± 2.6 ^c^	14.3 ± 2.0 ^c^	6.1 ± 1.2 ^d^	7.2 ± 1.0 ^d^	Aroma of wine, fruit, and flowers
Ethyl cinnamate	0.017	8.1 ± 1.5 ^e^	16.4 ± 1.4 ^d^	41.3 ± 2.3 ^a^	34.0 ± 2.9 ^b^	13.2 ± 2.3 ^d^	29.2 ± 2.3 ^c^	Cinnamon and strawberry aromas
γ-Decalactone	1.1 × 10^−3^	265.2 ± 9.5 ^a^	276.8 ± 18.2 ^a^	216.1 ± 9.8 ^b^	249.0 ± 27.3 ^ab^	147.1 ± 9.2 ^c^	298.2 ± 27.2 ^a^	Coconut and peach aromas
Phenylethyl alcohol	0.564	806.2 ± 8.3 ^c^	1075.1 ± 14.3 ^a^	1024.1 ± 17.5 ^b^	1102.2 ± 19.8 ^a^	575.3 ± 10.1 ^d^	66.0 ± 6.2 ^e^	Rose fragrance
3-Methyl-1-butanol	0.04	2605.1 ± 133.3 ^c^	2063.0 ± 58.2 ^e^	5505 ± 158.3 ^a^	2878 ± 106.3 ^b^	2266 ± 105.6 ^d^	899 ± 56.6 ^f^	Apple brandy fragrance
2-Methyl-1-butanol	0.0159	3817.2 ± 199.5 ^b^	2786.1 ± 173.3 ^c^	5671.3 ± 243.7 ^a^	3878.4 ± 171.4 ^b^	2746.8 ± 217.8 ^c^	2033.8 ± 112.6 ^d^	Alcohol flavor
2-Methyl-1-propanol	6.506	2.0 ± 0.2 ^d^	6.4 ± 0.2 ^a^	5.1 ± 0.2 ^b^	4.1 ± 0.2 ^c^	2.2 ± 0.1 ^d^	4.0 ± 0.1 ^c^	Special odor
1-Nonanol	0.045	178.3 ± 21.3 ^b^	150.3 ± 16.5 ^c^	114.7 ± 8.7 ^d^	285.3 ± 19.8 ^a^	82.8 ± 9.5 ^e^	131.3 ± 18.4 ^d^	Rose wax, fruity aroma
Geraniol	0.007	546.3 ± 37.3 ^b^	672.4 ± 26.5 ^a^	286.2 ± 54.2 ^c^	592.7 ± 90.4 ^b^	167.7 ± 56.7 ^d^	196.1 ± 39.6 ^d^	Rose gas
1-Hexanol	0.006	282.1 ± 17.6 ^c^	326.8 ± 28.7 ^b^	190.3 ± 57.8 ^d^	744.5 ± 45.4 ^a^	100.1 ± 13.3 ^e^	750.0 ± 40.5 ^a^	Fragrance of branches and leaves, fragrance of fat
Linalool	2.2 × 10^−4^	5543.3 ± 91.5 ^d^	10,661.1 ± 136.7 ^b^	12,934.0 ± 273.8 ^a^	6691.2 ± 136.8 ^c^	6656.3 ± 91.8 ^c^	3825.4 ± 182.2 ^e^	Fragrant lemon fragrance
2-Nonanol	0.058	24.1 ± 2.2 ^c^	52.4 ± 3.1 ^a^	10.4 ± 1.5 ^d^	31.2 ± 2.5 ^b^	2.2 ± 0.3 ^e^	11.4 ± 0.3 ^d^	Citrus and cheese aroma
Citronellol	0.062	25.3 ± 0.3 ^b^	30.3 ± 1.1 ^a^	21.2 ± 1.8 ^c^	32.0 ± 1.9 ^a^	11.2 ± 0.8 ^e^	15.4 ± 0.6 ^d^	Rose fragrance
1-Dodecanol	0.016	122.2 ± 2.1 ^b^	104.1 ± 4.2 ^c^	122.0 ± 5.8 ^b^	163.1 ± 6.7 ^a^	52.3 ± 3.3 ^e^	96.2 ± 6.1 ^d^	Pungent odor
3-Methyl-1-pentanol	7.5 × 10^−3^	11.1 ± 1.5 ^c^	16.0 ± 3.7 ^b^	14.3 ± 1.3 ^c^	24.4 ± 4.6 ^a^	14.0 ± 3.8 ^c^	21.1 ± 4.2 ^a^	Fruit flavor and wine aroma
1-Heptanol	5.4 × 10^−3^	133.2 ± 9.2 ^a^	115.8 ± 11.4 ^b^	75.4 ± 4.6 ^c^	121.2 ± 7.1 ^b^	37.1 ± 4.2 ^c^	112.0 ± 6.6 ^b^	Oil and citrus aromas
1-Octen-3-ol	1.5 × 10^−3^	337.3 ± 20 ^b^	2117.1 ± 60.3 ^a^	245.0 ± 20.3 ^c^	327.7 ± 33.4 ^b^	96.3 ± 7.6 ^d^	102.8 ± 7.6 ^d^	Fresh chicken fragrance
(E)-2-Octen-1-ol	0.02	9.0 ± 1.2 ^b^	10.1 ± 2.4 ^b^	10.3 ± 1.3 ^b^	18.9 ± 1.3 ^a^	7.2 ± 0.5 ^c^	5.1 ± 0.5 ^d^	Citrus aroma
Toluene	0.527	10.2 ± 1.6 ^c^	5.1 ± 0.5 ^e^	8.4 ± 0.4 ^d^	12.3 ± 0.6 ^b^	3.2 ± 0.5 ^f^	15.1 ± 1.3 ^a^	Aromatic taste
Naphthalene	0.006	182.3 ± 20.3 ^a^	107.3 ± 10.3 ^b^	102.1 ± 7.4 ^b^	167.6 ± 13.2 ^a^	72.4 ± 5.3 ^d^	92.3 ± 5.4 ^c^	tarry
Styrene	0.065	7.1 ± 0.5 ^c^	6.2 ± 0.6 ^cd^	5.0 ± 0.5 ^d^	8.1 ± 0.3 ^b^	2.0 ± 0.3 ^e^	10.8 ± 0.5 ^a^	Aromatic odor
Myrcene	1.2 × 10^−3^	177.1 ± 17.3 ^b^	166.2 ± 17.3 ^bc^	147.1 ± 8.6 ^c^	268.9 ± 25.5 ^a^	107.0 ± 17.2 ^d^	183.2 ± 8.2 ^b^	Fragrance gas
(E)-β-Ocimene	0.034	2.9 ± 0.3 ^a^	3.2 ± 0.3 ^a^	2.2 ± 0.1 ^b^	3.0 ± 0.3 ^a^	2.1 ± 0.1 ^b^	2.1 ± 0.2 ^b^	medicinal
Decanal	0.003	2244.2 ± 113.4 ^b^	1994.1 ± 247.3 ^c^	1596.1 ± 100.2 ^d^	3594.2 ± 300.1 ^a^	1353.6 ± 77.2 ^e^	1082.3 ± 183.4 ^f^	Lemon oil fragrance
Nonanal	1.1 × 10^−3^	3197.2 ± 91.3 ^c^	2922.4 ± 109.1 ^d^	4301.4 ± 191.1 ^b^	5327.1 ± 200.1 ^a^	2382.0 ± 155.2 ^e^	1275.1 ± 91.3 ^f^	Honey wax floral fragrance
Benzeneacetaldehyde	6.3 × 10^−3^	285.1 ± 25.3 ^a^	151.3 ± 14.2 ^b^	115.4 ± 6.4 ^c^	256.6 ± 16.6 ^a^	108.2 ± 11.5 ^c^	39.2 ± 8.2 ^d^	Fragrance of hyacinth and narcissus flowers
β-Cyclocitral	0.003	125.3 ± 10.3 ^b^	498.1 ± 13.2 ^a^	69.1 ± 3.1 ^c^	125.2 ± 7.3 ^b^	46.3 ± 3.2 ^d^	116.2 ± 10.3 ^b^	Damascus ketone fragrance
(E)-3-Heptylacrolein	0.017	8.9 ± 0.6 ^a^	5.2 ± 0.6 ^b^	4.3 ± 0.6 ^c^	5.2 ± 0.6 ^b^	2.0 ± 0.6 ^cd^	2.1 ± 0.3 ^d^	Fat fragrance, earthy fragrance
Undecanal	0.012	24.2 ± 0.8 ^b^	19.0 ± 0.8 ^c^	13.1 ± 0.8 ^e^	28.7 ± 1.9 ^a^	15.2 ± 0.8 ^d^	10.2 ± 0.8 ^f^	Rose and wax fragrance
Undecan-4-olide	0.002	58.3 ± 5.1 ^b^	59.1 ± 5.1 ^b^	49.2 ± 5.1 ^b^	79.2 ± 10.3 ^a^	52.1 ± 10.3 ^b^	83.2 ± 10.3 ^a^	Peach aroma
Heptanal	0.004	42.1 ± 3.2 ^b^	26.0 ± 3.2 ^c^	19.2 ± 5.6 ^d^	49.3 ± 5.6 ^a^	24.2 ± 3.2 ^c^	19.3 ± 3.2 ^d^	Fruity aroma
2-Undecanone	0.005	17.3 ± 2.2 ^c^	25.4 ± 4.4 ^b^	7.0 ± 2.2 ^d^	36.7 ± 8.3 ^a^	4.2 ± 1.5 ^d^	6.2 ± 1.5 ^d^	Citrus aroma, oil aroma
1-Octen-3-one	3 × 10^−6^	32,923.2 ± 3333.3 ^d^	26,824.3 ± 3333.3 ^b^	29,481.6 ± 6666.6 ^b^	67,959.1 ± 3333.3 ^a^	15,416.3 ± 3333.3 ^c^	12,037.7 ± 3333.3 ^c^	Creamy
β-Lonone	7 × 10^−6^	8332.2 ± 1429.7 ^e^	42,471.2 ± 4286.6 ^d^	118,625.4 ± 5714.8 ^a^	110,095.4 ± 4286.3 ^a^	55,696.3 ± 2857.4 ^c^	95,046.4 ± 1429.7 ^b^	Violet fragrance
4-Hydroxy-3-methoxystyrene	0.012	24.1 ± 0.8 ^b^	30.4 ± 1.7 ^a^	6.3 ± 0.8 ^d^	12.4 ± 0.8 ^c^	5.2 ± 0.8 ^d^	10.1 ± 1.7 ^c^	Spices and cloves
Eugenol	7.1 × 10^−4^	31.4 ± 7.3 ^d^	119.1 ± 14.8 ^b^	57 ± 0.314.2 ^c^	185.6 ± 28.4 ^a^	33.4 ± 9.4 ^d^	81.1 ± 14.2 ^bc^	Flower fragrance, clove oil fragrance

Note: According to Tukey’s test, there was no significant difference in the data representation of the same letter in the same row. The flavor characteristic descriptions are sourced from https://pubchem.ncbi.nlm.nih.gov and https://www.chemicalbook.com (accessed on 1 May 2024.).

## Data Availability

All relevant data are within the paper.
